# The Impact of Aging on Meniscal Tears and Chondral Lesions in Men: Insights from First-Time Arthroscopic Knee Evaluation

**DOI:** 10.3390/life15081305

**Published:** 2025-08-17

**Authors:** Sorin Florescu, Cristian Zaharia, George Andrei Drăghici, Dragoş Vasile Nica, Cosmin Grațian Damian

**Affiliations:** 1 Discipline of Orthopedics-Traumatology, Department XV, “Victor Babes” University of Medicine and Pharmacy of Timisoara, Piața Eftimie Murgu 2, 300041 Timisoara, Romania; sorin.florescu@umft.ro; 2 Faculty of Dental Medicine, “Victor Babes” University of Medicine and Pharmacy of Timisoara, 2 Eftimie Murgu Square, 300041 Timisoara, Romania; 3 Research Center in Dental Medicine Using Conventional and Alternative Technologies, Department of Prostheses Technology and Dental Materials, Faculty of Dental Medicine, “Victor Babes” University of Medicine and Pharmacy of Timisoara, 9 Revolutiei 1989 Ave., 300070 Timisoara, Romania; 4 Research Center for Pharmaco-Toxicological Evaluations, Faculty of Pharmacy, “Victor Babes” University of Medicine and Pharmacy of Timisoara, Eftimie Murgu Square No. 2, 300041 Timisoara, Romania; nicadragos@gmail.com; 5 Faculty of Pharmacy, “Victor Babes” University of Medicine and Pharmacy of Timisoara, Eftimie Murgu Square No. 2, 300041 Timisoara, Romania; 6 The National Institute of Research—Development for Machines and Installations Designed for Agriculture and Food Industry (INMA), Bulevardul Ion Ionescu de la Brad 6, 077190 București, Romania; 7 Faculty of Medicine, “Vasile Goldiș” Western University of Arad, Bulevardul Revoluției 94, 310025 Arad, Romania; damian.gratian@uvvg.ro

**Keywords:** menisci, knee, cartilage, articular/injuries, aging, arthroscopy, patellofemoral joint, male

## Abstract

(1) Background: This is the first study investigating the age-related distribution of meniscal and chondral lesions in an all-male cohort undergoing first-time knee arthroscopy. (2) Methods: The study population included 876 adult men stratified into five decade-based age groups. Lesions were confirmed arthroscopically after MRI evaluation, with chondral injuries being graded using the ICRS system. (3) Results: The frequency of medial meniscal tears differed significantly across age strata (*p* = 0.042), increasing with age. No differences were detected for lateral meniscal damage or patellar damage. Age was a significant predictor of medial meniscal damage (OR = 1.04; *p* = 0.003), but not for other types of knee injuries. Medial meniscal damage correlated with patellar damage (men < 30, 50–59) and inversely with lateral damage (30–39); other correlations were non-significant. Chondropathy severity increased significantly with age in the medial femoral condyle and medial tibial plateau (*p* < 0.001), with severe (ICRS grade IV) lesions showing a steep rise after 60 years. Cartilage lesions at the level of the lateral knee compartment were, by contrast, less prevalent and less severe, with no significant variation across age groups. (4) Conclusions: These findings demonstrate that intra-articular knee pathology in men shifts with age, with medial compartment degeneration becoming increasingly prominent.

## 1. Introduction

Meniscal tears and chondral (cartilage) lesions rank among the most common knee injuries encountered in orthopedic medicine [1]. The etiology of meniscal injuries differs with age [2]. Acute, traumatic meniscal tears typically affect young, active individuals during sports, peaking in the third and fourth decades of life [3]. In contrast, degenerative meniscal tears become prevalent as people age, resulting from repetitive mechanical loading and age-related tissue dehydration [1,4]. These lesions account for roughly 30% of all meniscus tears, peaking in incidence around 40–50 years of age in men [5,6,7]. Notably, the pattern of meniscal tear also shifts with age: lateral meniscus tears are more likely in younger patients (often due to acute trauma), whereas medial meniscus tears increase in frequency with advancing age [8,9]. These age-related differences in meniscal lesion patterns are clinically important for diagnosis and management.

Articular cartilage lesions (chondral injuries) of the knee are also closely associated with age [10]. Focal chondral defects occur in young patients primarily due to acute trauma (e.g., osteochondral fractures from sports injuries or patellar dislocations). In such cases, the rest of the joint cartilage is typically healthy, and the lesion is isolated [11]. After the fourth decade of life, however, degenerative changes in cartilage become common. In fact, osteoarthritis is the leading cause of chondral lesions in patients over 40 years [12]. Furthermore, older patients tend to have multiple chondral lesions, whereas younger patients more often have a single isolated defect [13]. Since the meniscus and cartilage are functionally interrelated, their injuries often co-occur: loss of meniscal integrity can accelerate cartilage wear, and conversely, chronic cartilage degeneration often accompanies meniscal tears in older adults. Older patients, hence, tend to exhibit combined meniscal-chondral injuries rather than isolated structural damage [14,15,16].

Lesions of the patellofemoral compartment deserve special attention. Patellofemoral chondral lesions are a unique subset that can cause anterior knee pain and are notoriously difficult to treat. Patellar lesions can occur at both extremes of age: younger individuals may experience isolated patellar cartilage defects due to injury [17], while older individuals may develop patellofemoral arthritic changes as part of generalized knee degeneration [18]. Understanding how age affects the occurrence of patellar lesions—in addition to meniscal and other chondral injuries—can guide clinicians in forming differential diagnoses for knee pain and mechanical symptoms across different age groups.

Despite these known general patterns, there is relatively limited quantitative data directly comparing the rates of meniscal vs. chondral lesions by age in a broad series of arthroscopies. Data obtained exclusively from first-time arthroscopic interventions are scarce. Most previous studies have focused on specific subgroups, e.g., meniscal tears in isolation [19,20,21,22] or cartilage lesions in the setting of ligament injuries [23,24,25]. In a real-world arthroscopic practice, patients present with a spectrum of intra-articular pathology that may include meniscal tears, cartilage lesions, or both. The influence of age on this spectrum has implications for clinical decision-making—for example, whether an older patient’s knee symptoms are more likely due to degenerative arthritis rather than a discrete tear that could be addressed surgically. This observational study was designed to explore how structural knee lesions evolve with age in male patients undergoing first-time arthroscopic surgery. Rather than testing a narrow hypothesis, our investigation was driven by the broader premise that aging plays a significant role in shaping the pattern and severity of intra-articular knee damage. By stratifying patients into age-based groups and examining meniscal, patellofemoral, and femorotibial cartilage lesions in parallel, we aimed to clarify age-related trends that may influence diagnostic reasoning and operative decision-making in routine orthopedic practice. The delineation of these patterns provides insights that can help clinicians anticipate the likely pathology based on patient age and tailor management accordingly.

## 2. Materials and Methods

### 2.1. Study Population

The study population included male patients admitted to the Arad County Emergency Hospital (SCJU Arad) for knee arthroscopic procedures between March 2013 and March 2025. This study was conducted at the Clinic of Orthopaedic and Traumatology. This clinic performs over 300 knee arthroscopies each year [26], and hence, it provides a robust clinical base for analyzing intra-articular knee pathology across age groups. Ethical approval was obtained from the Institutional Ethics Committee of the aforementioned hospital (approval number 92/20.01.2025).

A key reason behind this targeted recruitment strategy is that men are more often involved in activities that increase the risk of meniscal and chondral wear, including physically demanding occupations and sports [1]. Moreover, orthopedic literature has primarily addressed mixed populations without allowing for accurate insights into age-related degenerative trends in the absence of sex-specific injury biases. The inclusion of patients with first-time arthroscopy ensures unaltered knee anatomy and pathology. The collected variables included age, the type of meniscal tear, the presence of associated patellar lesions, and the type and severity of co-existing chondropathy. These parameters were selected based on their relevance to injury mechanisms and long-term prognostic expectations [27]. Other determinants such as comorbidities, body mass index (BMI), physical activity level, prior knee injuries, and occupational factors were not taken into account in our investigation. These variables are typically incomplete or inconsistently available in retrospective datasets [28], and their use can dilute the clarity of the age-based comparisons, lead to model overfitting, and reduce statistical power [29].

Male patients were referred for arthroscopy following persistent knee symptoms (e.g., pain, locking, or catching) that did not improve after conservative treatment, such as physiotherapy, nonsteroidal anti-inflammatory drugs (NSAIDs), or intra-articular injections. As part of standard clinical evaluation, all patients first underwent preoperative magnetic resonance imaging (MRI). These analyses were reviewed by orthopedic surgeons to identify suspected meniscal and cartilage abnormalities. The definitive evaluation was performed intraoperatively during arthroscopy. This aimed to validate the diagnosis and guide therapeutic measures. All meniscal tears were directly visualized, classified according to location (medial or lateral), and documented in operative reports. The presence of associated patellar lesions was also noted. Chondral lesions were evaluated using direct arthroscopic inspection and graded as per the International Cartilage Repair Society (ICRS) classification, with grades ranging from 0, i.e., normal cartilage, to IV, i.e., partial loss of cartilage thickness, defects extending >50% of cartilage depth, and possibly reaching the calcified layer [30]. Each cartilage surface was examined separately, including the medial and lateral femoral condyles, tibial plateaus, and patellofemoral joint. The final dataset used for analysis was compiled from operative notes, surgical video recordings when available, and preoperative imaging reports. Only lesions confirmed during arthroscopy were included in the statistical evaluation.

The study population was stratified into five decade-based age groups: <30, 30–39, 40–49, 50–59, and ≥60 years. Patients under 30 years are the most active population, with a higher prevalence of traumatic injuries. The 30–39 and 40–49 groups cover the decades where early degenerative changes begin to emerge—often overlapping with high physical activity levels. The 50–59 decade marks the onset of more advanced degenerative joint disease in most males. Patients 60 years and older, referred to as elderly patients, in whom cartilage loss and meniscal wear are multifactorial and clinically relevant [1,29]. This stratification approach hence captures key physiological and clinical transitions relevant to musculoskeletal health in male patients.

The primary inclusion criteria were as follows: (*i*) adult men (≥18 years) undergoing first-time knee arthroscopy; (*ii*) presence of meniscal tears (and associated chondral lesions) confirmed intraoperatively or via preoperative MRI; (*iii*) documented symptoms of knee pain, mechanical dysfunction (locking, catching), or swelling for at least 6 weeks; (*iv*) failure of conservative treatment (e.g., NSAIDs, physiotherapy, intra-articular injections) for longer than 6 weeks; and (*v*) available informed consent for data usage in research. Exclusion criteria included the following: (*i*) history of previous ipsilateral knee arthroscopy or major knee surgery, including reconstruction of anterior cruciate ligament (ACL), meniscectomy, osteotomy, and total knee arthroplasty; (*ii*) acute traumatic meniscus injuries (e.g., sports injury, high-energy trauma) within the past 3 months; (*iii*) diagnosis of autoimmune/inflammatory arthropathies (e.g., rheumatoid arthritis, psoriatic arthritis, lupus); (*iv*) history of septic arthritis or primary/metastatic bone tumors affecting the knee; (*v*) missing or poor-quality MRI/arthroscopy reports; (*vi*) concurrent neuromuscular disorders (e.g., Parkinson’s disease, stroke) affecting gait or joint loading; and (*vii*) bilateral knee pathology.

Given the retrospective nature of the dataset and variability in clinical documentation, we were unable to systematically classify meniscal or chondral lesions as traumatic or degenerative. Lesion etiology was therefore not included in the analysis, with all cases being evaluated based on anatomical location and severity alone. All MRI evaluations were interpreted by orthopedic surgeons involved in the surgical care of the patients. Lesion classification and grading were confirmed intraoperatively by the attending arthroscopist during the procedure. While formal interobserver agreement analysis was not conducted, the consistent involvement of senior orthopedic surgeons in both preoperative and intraoperative evaluation served to minimize classification variability.

This was a retrospective observational study. All patient data were initially extracted from existing medical records. The ethics approval issued in January 2025 covered the full retrospective review of cases operated between March 2013 and March 2025. As part of the approved protocol, researchers then contacted all eligible patients to obtain specific retrospective informed consent for the use of their de-identified medical information. Only data from patients who voluntarily provided this explicit written consent were included in this study’s analysis.

### 2.2. Statistical Analysis

We first applied Pearson’s Chi-square tests to identify differences in the prevalence of medial meniscal tears, lateral meniscal tears, and patellar lesions among different age strata. This procedure aimed to determine unadjusted associations of age with these variables, allowing us to distinguish crude relationships from adjusted effects and ensure an unbiased selection of variables for inclusion in the logistic regression model [31,32]. All these metrics were included in the logistic regression models irrespective of their univariate statistical significance. This approach ensured that adjusted associations are not biased by the omission of potentially important confounders. For regression analysis, the dependent variables were the presence of medial meniscus damage, the presence of lateral meniscus damage, and the presence of patellar damage. Each variable was coded in a binary format, with “yes” indicating that the patient had arthroscopically confirmed medial meniscus damage and “no” indicating the absence of such damage. The independent variable was age, which was treated as a continuous predictor. Phi coefficients were then used to measure the strength of association between medial meniscal damage, lateral meniscal damage, and associated patellar damage across different age categories [28]. Finally, we again applied frequency analysis (using Pearson’s Chi-square tests) to determine how cartilage lesion severity is distributed across age groups. Statistical significance was set at *p* < 0.05 [32].

## 3. Results

Table 1 shows the frequency of meniscal tears and associated patellar damage across different age groups. A significant **age-related effect was observed for** medial meniscus pathology (Chi-square test, *p* = 0.042), with its prevalence tending to increase progressively with advancing age. Lateral meniscus damage was less prevalent, and no significant differences existed between different age groups (Chi-square test, *p* = 0.634). The distribution of co-existing patellar lesions also did not differ among strata (Chi-square test, *p* = 0.113).

Table 2 presents the results of logistic regression analyses examining the relationship between age and meniscal/patellar injuries. Age was significantly associated with medial meniscal damage, with each additional year increasing the odds by approximately 3%. In contrast, age did not emerge as a predictor of lateral meniscal damage or patellar damage.

Table 3 reveals intra-strata associations between different types of knee injuries. Medial meniscal damage was directly associated with any patellar damage in men younger than 30 years. Male patients between the ages of 50 and 59 exhibited a comparable, yet stronger, relationship. We also identified a significant inverse correlation between medial meniscal damage and lateral meniscal damage in the 30–39-year-old cohort. In contrast, the other associations were not significant.

Table 4 presents the distribution of femoral chondropathy severity classes across age groups. In the case of chondropathy on the medial femoral condyle, we identified significant inter-strata differences regarding the distribution of ICRS scores (Chi-square test, *p* < 0.001). The severity of cartilage lesions increased progressively with age, with grade IV lesions becoming the dominant pattern after age 50 and particularly pronounced in patients aged 60 years and older (Table 3). However, no significant inter-strata differences in ICRS score distribution were observed for chondral lesions at the lateral femoral condyle (Chi-square test, *p* = 0.053). The cartilage of these femoral structures remained largely preserved in most age groups, including older adults. Grade 4 lesions were rare, only appearing in a minority of males 40 years and above (Table 3).

The distribution of tibial chondropathy severity classes across age categories is given in Table 5. The severity of cartilage lesions at this site varied significantly with age (Chi-square test, *p* < 0.001). There was an evident age-associated trend of cartilage deterioration in the medial tibial plateau, with mild changes beginning in the 30 s and severe chondropathy (ICRS grade IV) increasing abruptly and dominating in patients 60 years and above. Interestingly, severe chondropathy appeared at later ages than in the case of the medial femoral condyle. We note that the lateral tibial plateau was less affected by age than the medial side (Chi-square test, *p* = 0.260). Thus, over 80% of patients still displayed normal cartilage even in middle age, with severe degeneration being rare across all age groups.

## 4. Discussion

To our knowledge, this is the first large-scale comprehensive analysis examining the distribution and severity of meniscal and chondral lesions in an exclusively male population undergoing first-time knee arthroscopy. It uses a unique decade-based age stratification (from <30 to ≥60 years) combined with correlation analysis across medial meniscus, lateral meniscus, and patellofemoral lesions. This level of granular, comparative mapping has not been reported before and reveals age-specific inter-lesion dynamics. We identified an earlier-than-expected onset of medial compartment cartilage degeneration (even in men under 40 years) and demonstrated how meniscal and chondral injuries tend to co-occur and intensify with age—a progression that has been described anecdotally but not quantified this precisely in the literature. These data improve the current understanding of the natural history of knee injuries.

It is well-established that meniscal tears constitute one of the most prevalent intra-articular knee pathologies, with an estimated prevalence of 12–14% in adult populations [33,34]. Articular cartilage lesions often co-occur in knees requiring arthroscopy—large series have reported chondral damage in 60–66% of knee arthroscopies [35]. Our results delineate clear trends: medial meniscal tears became more prevalent with advancing age, whereas lateral meniscal tears remained relatively stable across age groups. Chondral lesions (knee chondropathy) were common and climbed sharply with age, affecting a large proportion of patients by their early 40 s in our cohort. These findings reveal that even first-time knee injuries in older males often involve a combination of meniscal and cartilage pathology.

The selective age-related increase in the prevalence of medial meniscus tears is consistent with orthopedic data. After investigating 782 patients undergoing meniscal procedures, Ridley et al. found that isolated medial tears become more prevalent in older individuals; these lesions predominated in patients older than 30 years [9]. In a cohort study with 1855 patients, Çolak et al. reported that the odds of having medial meniscal tears almost double with each decade of aging [2]. Taunton et al. analyzed 2002 running injuries and identified lower age as a protective factor against meniscal injury [36]. Not only is the medial meniscus typically affected in individuals aged over 40 years, but more than one-third of cases also display multiple tears [37].

In our cohort, lateral meniscal tears were less frequent than medial meniscal tears, irrespective of age strata. This trend is congruent with prior evidence. Thus, Ridley et al. found isolated lateral meniscal injuries in 21.7% of patients over 30 years, whereas isolated medial meniscal lesions occurred in 52.2% [9]. Stoker reported a 2.5-fold higher incidence of medial meniscal damage vs. lateral meniscal damage [38]. In a study with 830 patients with injuries of the anterior cruciate ligament (ACL), Nakamae et al. also observed a higher frequency of medial meniscal tears—32.0% vs. 26.5% [39]. These differences may stem from the physiological particularities of the two menisci. The lateral meniscus is more mobile because of looser capsular attachments, lack of fixation to the lateral collateral ligament, posterior stabilization by meniscofemoral ligaments rather than rigid tibial anchoring, and dynamic interaction with the popliteus tendon. This greater mobility protects the lateral meniscus from degenerative tears but makes it more susceptible to certain acute injuries (e.g., discoid meniscus, bucket-handle tears) [40,41,42]. These features could also explain why the lateral meniscus appears less affected by aging. In fact, lateral meniscus tears are relatively less common in older individuals but are seen more often in younger, active individuals due to high-impact traumatic injuries (often with ACL tears) and the presence of a discoid meniscus [41].

Our results support the well-established clinical observation that the medial compartment of the knee bears significantly more load than the lateral compartment, predisposing it to cartilage degeneration (chondropathy) and osteoarthritis [43,44]. Age exerted a significant effect on the prevalence of chondral lesions at the medial femoral condyle, with ICRS Grade II lesions and even some ICRS Grade III/IV lesions being observed in relatively young strata—male patients under 30 years and the 30–39-year group. This articular cartilage may hence begin to succumb to wear and micro-injury earlier than the menisci. Its avascular nature and limited healing capacity likely contributed to this outcome: once adulthood is reached and skeletal maturation is complete, cartilage repair potential declines, making accumulated damage more likely to persist [43,45].

We identified an earlier onset of severe chondropathy at the medial femoral condyle vs. the medial tibial plateau. Intrinsic differences in biomechanical loading and joint anatomy may help explain these findings. The femoral condyle is prone to earlier focal cartilage damage since its convex shape concentrates mechanical stress during knee flexion. The relatively flat tibial plateau, by contrast, distributes load more evenly, being more protected from severe degeneration [46,47]. On the other hand, the medial meniscus absorbs shocks and redistributes loads for the tibial plateau, delaying its involvement [48]. This protective mechanism, however, may diminish with age as meniscal degeneration progresses, yielding increased mechanical stress on the tibial cartilage and the subsequent emergence of more advanced lesions at later stages [2,47,48]. Differences in subchondral bone remodeling may also be involved, with the femoral condyle exhibiting earlier adaptive changes in response to microdamage and the tibial plateau demonstrating a more gradual progression toward full-thickness chondral loss.

Both the medial femoral condyle and the medial plateau showed a progressive increase in ICRS Grade IV full-thickness chondral lesions with age, accelerating after age 60 years. This non-linear pattern of degeneration aligns with current medical evidence [49]. Morphological deterioration of the articular cartilage manifests as quantifiable changes in joint biomechanics, detectable via vibroarthrography (VAG)—a non-invasive technique that records the sounds and vibrations produced by joints during motion. Using such an approach, Kręcisz et al. reported that parameters of vibroarthrographic signals, such as recurrence rate and multi-scale entropy, decrease with age, reflecting progressive degenerative changes in knee joint motion [50].

In our cohort, the prevalence of patellar damage appeared to be independent of the effect of aging. Although this effect seems counterintuitive, there is evidence to support our data. For example, Ding et al. reported no effect of age on patellar cartilage volume in males after adjustment for body height, weight, and bone size. However, sex differences in cartilage volume were more pronounced in those aged 50 years and older [51]. For the same gender, Değirmenci et al. found no significant association between age and patella height as determined using the Blumensaat line measurement [52]. Similarly, Hudelmaier et al. reported a non-significant reduction in patellar cartilage thickness (−6%) in men aged 20–30 years and subjects aged 50–78 years [53]. It is plausible that various factors, such as physical activity and BMI, may play a more critical role in the health of patellar cartilage than age alone [52].

Medial meniscal damage is associated directly with patellar damage in men under 30 years. The co-occurrence of these knee pathologies may reflect shared injury mechanisms, such as high-impact sports or concurrent trauma to both structures [1]. Male patients aged 50 to 59 years exhibited a comparable but more robust relationship. Age-related degeneration may account for this association—the breakdown of the medial meniscus leads to altered joint mechanics and subsequent patellar cartilage damage. The significant inverse correlation between medial and lateral meniscal damage among individuals aged 30 to 39 years, on the other hand, indicates a potential divergence in injury mechanisms or loading patterns in this age group. For instance, unilateral loading, occupational factors, or previous injuries might shift biomechanical stresses to a particular compartment of the knee [42]. However, we note that most age groups showed no statistically significant associations between different types of knee injuries. This implies that the patterns of meniscal and patellar damage may be more variable or influenced by other unmeasured factors, including degenerative changes, prevalence of asymptomatic conditions, BMI, physical activity level, or previous surgical history [1,2,6]. In fact, many factors affect compartment-specific joint loading and osteoarthritic progression. Thus, varus alignment tends to increase mechanical stress on the medial compartment, accelerating chondral wear and degenerative changes, while valgus alignment predominantly affects the lateral side. These biomechanical factors may partially explain the greater severity and earlier onset of medial chondropathy observed in our cohort. This concept is supported by previous studies; for example, Al-Bayati et al. identified postural dysfunction of the foot as a driver of osteoarthritis development and progression [54].

From a practical point of view, these present findings carry several implications for clinical decision-making when evaluating meniscal tears and cartilage injuries. For a young male patient (e.g., in his twenties) presenting with an acute knee injury, one might expect a higher likelihood of an isolated meniscus tear (often lateral) without extensive chondral damage. The priority in such cases may be to repair and preserve the meniscus and to address any concurrent ligament injuries, thereby restoring stability and preventing early-onset degeneration [55]. Our data reinforce this pro-intervention approach in the young: because cartilage changes can begin soon after a meniscal injury, prompt repair of meniscal tears (and stabilization of the knee if needed) could help mitigate the “downstream” chondral damage that seems to accumulate by the late 20 s. On the other hand, an older patient undergoing first-time arthroscopy for a meniscal tear is very likely to have co-existing articular cartilage lesions—in our series, most men above 40 years had some degree of chondropathy. Therefore, arthroscopic management in middle-aged and older men often needs to address both the meniscus and cartilage. Moreover, our findings underscore that a 50-year-old male with a torn medial meniscus likely does not have a pristine joint—rather, the tear may be the “tip of the iceberg” of broader degenerative changes [56].

This study contributes several novel insights to the current understanding of knee injury patterns. First, we identified an earlier-than-expected onset of chondral pathology in male patients. The presence of osteochondral damage by the third decade of life—while patients are still relatively young and active—remains underreported in previous studies. Second, stratification into age quartiles and examination of the co-occurrence of meniscal and chondral injuries allowed us to demonstrate how these pathologies intersect over time. Our results revealed that combined meniscal tear and cartilage lesion cases became more frequent with age. To our knowledge, such a granular analysis of concurrent lesion patterns across ages is new. It contributes to the understanding that meniscal tears and cartilage degeneration are not isolated phenomena in older patients but rather tend to cluster as age-related comorbidities of the knee [57]. Third, we focus on a male patient population with first-time arthroscopies, thus obtaining a clear view of age effects without the confounding factor of sex. While female-specific patterns (possibly different rates of degenerative change) are not captured in our data—which we acknowledge as a limitation—the male-centric results fill a gap in knowledge for a demographic that comprises the majority of sports-related knee injury patients [58]. Finally, this study’s design—focusing on first-time interventions—delivers a clearer picture of the natural history of meniscal and chondral damage at presentation. This baseline can serve as a reference for future work on how subsequent surgeries or treatments alter the trajectory of knee joint degeneration.

The findings of the current study should be interpreted in the context of several limitations. Since our analysis was restricted to male patients, the present results cannot be extrapolated to females. Thus, female patients may exhibit different meniscal injury patterns or a different timeline for degenerative changes. Indeed, evidence suggests women have higher rates of knee osteoarthritis and possibly a greater risk of cartilage degeneration post-meniscus injury [59]. This retrospective study used only data extracted from the medical records of arthroscopy findings. This approach introduces potential biases, such as incomplete documentation or a lack of control over patients referred to surgery. Our focus on individuals undergoing first-time knee arthroscopy does not necessarily reflect the full natural history of meniscal or chondral lesions in the general male population. Rather, it shows the burden and distribution of symptomatic intra-articular pathology severe enough to require surgical intervention. In this context, many asymptomatic (or mildly symptomatic cases) likely fall outside the scope of this analysis since many of them go unreported or do not progress to surgery [60]. Nonetheless, one should consider that our objective was to identify age-related patterns in clinically significant, first-presenting knee injuries, not to determine their population-level prevalence. In addition, our analysis relied on arthroscopic confirmation—unlike imaging-based studies—allowing for direct visualization and grading of lesions and providing highly accurate and clinically meaningful findings [61]. Although limited in scope, this framework offers a high level of anatomical fidelity that population surveys cannot achieve. Moreover, subjects undergoing first-time arthroscopy reflect the endpoint of progressive, symptomatic degeneration. Studying this group helps clinicians understand which types of intra-articular damage are most likely to become clinically significant with age—key for patient care, prognosis, and surgical decisions.

Another drawback is the absence of etiological classification (traumatic vs. degenerative) for meniscal and patellar lesions. Although this distinction is clinically meaningful, it could not be reliably made from retrospective chart data, particularly for older patients or those without detailed injury histories. While we acknowledge the absence of detailed comorbidity data such as obesity, activity level, and limb alignment, the consistency of our findings across a sizeable cohort suggests that age-related degenerative patterns may be robust and observable even in the absence of such stratification. Moreover, several large-scale studies have demonstrated that aging itself is a strong independent risk factor for medial compartment degeneration, irrespective of modifiable risk factors like BMI or limb alignment [1,2,5,9,13]. While these variables could further refine the interpretation of individual cases, their absence does not fundamentally undermine the observed age-associated trends in meniscal or chondral injury patterns. Another limitation of this study is the absence of a formal inter-observer reliability analysis for MRI and arthroscopic grading. Although lesion classification was performed by senior orthopedic surgeons and verified during surgery, quantitative assessment of inter-rater agreement (e.g., kappa statistics) would improve reproducibility [62] and should be incorporated in future prospective studies.

One can also argue that exclusion of important biomechanical and lifestyle-related variables, including those mentioned above, weakens conclusions about causality and interindividual variability. On the other hand, their inclusion would involve extensive case exclusion since detailed data on BMI, alignment, and trauma history were inconsistently recorded across a 12-year dataset. In addition, this study was not designed to model patient-level risk but to describe structural lesion patterns across age strata. In fact, our approach aimed to identify typical presentations at surgical thresholds rather than make a causative prediction. Finally, as a single-center retrospective study, there is the possibility of observer bias in diagnosing lesions at arthroscopy. Different surgeons might have slightly varying thresholds for what constitutes a “lesion,” and subtle grade I changes in cartilage might be reported by one and not another. We attempted to minimize this by standardizing definitions and having experienced arthroscopists, but some inconsistency is inevitable. Despite these limitations, we believe the large sample and clear trends provide a valid overall picture, but the above factors should be kept in mind when applying our findings.

## 5. Conclusions

This study reveals clear age-related patterns in the presentation of meniscal and chondral lesions among men undergoing first-time knee arthroscopy. The prevalence of medial meniscal damage and medial compartment chondropathy increased significantly with age, while lateral and patellar damage patterns remained more stable and less severe across age groups. Notably, cartilage degeneration—particularly in the medial femoral condyle—began earlier than expected, with full-thickness lesions becoming predominant in patients over 60 years. The increasing co-occurrence of meniscal tears and cartilage lesions with advancing age highlights the need for comprehensive evaluation during arthroscopy and may inform targeted surgical strategies. While the findings are specific to a surgically treated, all-male population, they provide valuable clinical insight into the degenerative trajectory of the knee joint. The primary objective of this study was to elucidate age-related patterns of meniscal and chondral lesions in men undergoing first-time knee arthroscopy, thereby providing clinically relevant evidence to guide patient-specific management strategies and optimize surgical outcomes.

## Figures and Tables

**Table 1:** Distribution of meniscal tear types across different age groups.

Age Range	*n*	MMD		LMD		PTD	
		No	Yes	No	Yes	No	Yes
<30 years	348	168 (48.28%)	180 (51.72%)	191 (54.89%)	157 (45.11%)	270 (77.61%)	78 (22.39%)
30–39 years	194	78 (42.39%)	106 (57.61%)	105 (57.07%)	79 (42.93%)	140 (76.09%)	44 (23.91%)
40–49 years	86	36 (41.86%)	50 (58.14%)	52 (60.47%)	34 (39.53%)	80 (88.89%)	10 (11.11%)
50–59 years	128	48 (37.50%)	80 (62.50%)	72 (56.25%)	56 (43.75%)	110 (76.39%)	34 (23.61%)
≥60 years	120	40 (33.33%)	80 (66.67%)	75 (62.5%)	45 (37.5%)	90 (75.00%)	30 (25.00%)

MMD, medial meniscus damage; LMD, lateral meniscus damage; PTD, any patellar damage. The table reports the absolute number of cases, with percentages being listed in parentheses.

**Table 2:** Association between age and risk of meniscal and patellar injuries in male patients.

Variable	β	SE	Wald z	*p*-Value	OR (95% CI)
MMD	−0.03	0.01	2.98	0.003 **	1.03 (1.01–1.04)
LMD	0.02	0.011	−0.02	0.985	1.00 (0.98–1.02)
PTD	0.03	0.02	1.85	0.064	1.03 (0.98–1.07)

MMD, medial meniscus damage; LMD, lateral meniscus damage; PTD, any patellar damage; β, beta coefficient; SE, standard error; Wald z, Wald z-statistic; OR (95% CI), odds ratio with 95% confidence interval. Odds ratios are reported with the corresponding 95% confidence intervals in parentheses. Marked bold values (*) indicate significant differences (Wald test, ***—*p* < 0.001, **—*p* < 0.01, *—*p* < 0.05).

**Table 3:** Correlations among different types of knee joint injuries in age categories.

Variable	LMD	PTD
**<30 years**		
MMD	−0.06	0.25 ***
PTD	−0.08	
**30–39 years**		
MMD	−0.29 **	0.13
PTD	0.04	
**40–49 years**		
MMD	−0.25	0.11
PTD	0.10	
**50–59 years**		
MMD	0.21	0.40 *
PTD		0.15
**≥60 years**		
MMD	−0.31	0.20
PTD	0.44	

MMD, medial meniscus damage; LMD, lateral meniscus damage; PTD, any patellar damage. Marked values (*) indicate significant outcomes (Phi coefficients, ***—*p* < 0.001, **—*p* < 0.01, *—*p* < 0.05).

**Table 4:** Distribution of femoral chondropathy severity classes across age groups.

	Medial Femoral Condyle Chondropathy				
**Age Range**	0	1	2	3	4
<30 years	166 (47.7%)	14 (4.02%)	120 (34.48%)	32 (9.2%)	16 (4.6%)
30–39 years	80 (43.48%)	2 (1.09%)	76 (41.3%)	12 (6.52%)	14 (7.61%)
40–49 years	28 (32.56%)	2 (2.33%)	24 (27.91%)	4 (4.65%)	28 (32.56%)
50–59 years	24 (37.5%)	2 (6.25%)	10 (15.62%)	6 (9.38%)	20 (31.25%)
≥60 years	20 (16.67%)	0 (0.0%)	20 (16.67%)	0 (0.0%)	80 (66.66%)
	**Lateral Femoral Condyle Chondropathy**				
**Age Range**	0	1	2	3	4
<30 years	328 (94.25%)	0 (0.0%)	14 (4.02%)	4 (1.15%)	2(0.57%)
30–39 years	156 (84.78%)	0 (0.0%)	24 (13.04%)	4 (2.17%)	0 (0.0%)
40–49 years	72 (83.72%)	0 (0.0%)	4(4.65%)	2 (2.33%)	8 (9.3%)
50–59 years	56 (87.5%)	0 (0.0%)	6 (9.38%)	0 (0.0%)	2 (3.12%)
≥60 years	100 (83.33%)	0 (0.0%)	0 (0.0%)	0 (0.0%)	20 (16.67%)

0, ICRS grade 0 (normal cartilage, no damage); 1, ICRS grade I (superficial lesions, issues, cracks, and indentations); 2, ICRS grade II (fraying, lesions extending down to <50% of cartilage depth); 3, ICRS grade III (partial loss of cartilage thickness, cartilage defects extending down >50% of cartilage depth as well as down to the calcified layer); 4, ICRS grade IV (complete loss of cartilage thickness). The table reports the absolute number of cases, with percentages being listed in parentheses.

**Table 5:** Distribution of tibial chondropathy severity classes across age groups.

	Medial Tibial Plateau Chondropathy				
**Age range**	0	1	2	3	4
<30 years	264 (75.86%)	26 (7.47%)	42 (12.07%)	10 (2.87%)	6 (1.72%)
30–39 years	134 (72.83%)	2 (1.09%)	38 (20.65%)	4 (2.17%)	6 (3.26%)
40–49 years	50 (58.14%)	4 (4.65%)	10 (11.63%)	18(20.93%)	4 (4.65%)
50–59 years	34 (53.12%)	4 (6.25%)	8 (12.5%)	10 (15.62%)	8(12.5%)
≥60 years	4 (33.33%)	0 (0.0%)	0 (0.0%)	2 (16.67%)	6 (50.0%)
	**Lateral Tibial Plateau Chondropathy**				
**Age range**	0	1	2	3	4
<30 years	294 (84.48%)	10 (2.87%)	32 (9.2%)	10 (2.87%)	2 (0.57%)
30–39 years	150 (81.52%)	0 (0.0%)	26 (14.13%)	8 (4.35%)	0 (0.0%)
40–49 years	60 (69.77%)	4 (4.65%)	10 (11.63%)	10 (11.63%)	2 (2.33%)
50–59 years	52 (81.25%)	0 (0.0%)	8 (12.5%)	2 (3.12%)	2 (3.12%)
≥60 years	6 (50.0%)	0 (0.0%)	2 (16.67%)	2 (16.67%)	2 (16.67%)

0, ICRS grade 0 (normal cartilage, no damage); 1, ICRS grade I (superficial lesions, issues, cracks, and indentations); 2, ICRS grade II (fraying, lesions extending down to <50% of cartilage depth); 3, ICRS grade III (partial loss of cartilage thickness, cartilage defects extending down >50% of cartilage depth as well as down to the calcified layer); 4, ICRS grade IV (complete loss of cartilage thickness). The table reports the absolute number of cases, with percentages being listed in parentheses.

## Data Availability

All the data generated or analyzed in this study are included in this published article.

## Abbreviations

The following abbreviations are used in this manuscript:

ACL	Anterior cruciate ligament
BMI	Body mass index
CI	Confidence interval
ICRS	International Cartilage Repair Society
LMD	Lateral meniscus damage
MMD	medial meniscus damage
MRI	Magnetic resonance imaging
NSAID	Nonsteroidal anti-inflammatory drugs
OR	Odds ratio
PTD	Any patellar damage
SCJU	County Emergency Clinical Hospital
VAG	Vibroarthrography

## References

[1] Adams B.G., Houston M.N., Cameron K.L. (2021). The epidemiology of meniscus injury. Sports Med. Arthrosc. Rev..

[2] Snoeker B.A., Bakker E.W., Kegel C.A., Lucas C. (2013). Risk factors for meniscal tears: A systematic review including meta-analysis. J. Orthop. Sports Phys. Ther..

[3] Waldman S.D., Waldman S.D. (2024). Medial meniscal tear. Atlas of Common Pain Syndromes.

[4] Edmonds C.J., Foglia E., Booth P., Fu C.H., Gardner M. (2021). Dehydration in older people: A systematic review of the effects of dehydration on health outcomes, healthcare costs and cognitive performance. Arch. Gerontol. Geriatr..

[5] Englund M., Guermazi A., Gale D., Hunter D.J., Aliabadi P., Clancy M., Felson D.T. (2008). Incidental meniscal findings on knee MRI in middle-aged and elderly persons. N. Engl. J. Med..

[6] Englund M., Hulet C., Pereira H., Peretti G., Denti M. (2016). Degenerative meniscus lesions, cartilage degeneration, and osteoarthritis of the knee. Osteoarthritis: Diagnosis and Medical/Surgical Management.

[7] Pereira H., Cengiz I.F., Silva-Correia J., Oliveira J.M., Reis R.L., Espregueira-Mendes J., Randelli P., Dejour D., van Dijk C.N., Denti M., Seil R. (2017). The role of arthroscopy in the treatment of degenerative meniscus tear. Artroscopy.

[8] Khan H.I., Aitken D., Ding C., Blizzard L., Pelletier J.P., Martel-Pelletier J., Cicuttini F., Jones G. (2016). Natural history and clinical significance of meniscal tears over 8 years in a midlife cohort. BMC Musculoskelet. Disord..

[9] Ridley T.J., McCarthy M.A., Bollier M.J., Wolf B.R., Amendola A. (2017). Age differences in the prevalence of isolated medial and lateral meniscal tears in surgically treated patients. Iowa Orthop. J..

[10] Anwar W., Aziz Z., Rahman N., Khattak S.K., Ahmad I., Khan A.H. (2023). Arthroscopic evaluation of articular cartilage in knee injuries: A predictor of early osteoarthritis in young population. Prof. Med. J..

[11] Cole B.J., Burnett R.A., Kunze K.N., Tauro T., Chahla J., LaPrade R.F., Chahla J. (2020). Focal chondral injuries. Evidence-Based Management of Complex Knee Injuries.

[12] Jarecki J., Waśko M.K., Widuchowski W., Tomczyk-Warunek A., Wójciak M., Sowa I., Blicharski T. (2023). Knee cartilage lesion management—Current trends in clinical practice. J. Clin. Med..

[13] Widuchowski W., Widuchowski J., Trzaska T.J. (2007). Articular cartilage defects: Study of 25,124 knee arthroscopies. Knee.

[14] Valderrama J., Carredano X., León A., Vigueras C., Marín F., Acevedo M., Marin F. (2024). Prevalence of articular surface injuries in patients undergoing meniscal surgery: A retrospective analysis of 758 cases. Cureus.

[15] Ozeki N., Koga H., Sekiya I. (2022). Degenerative meniscus in knee osteoarthritis: From pathology to treatment. Life.

[16] Kutaish H., Klopfenstein A., Nasif Obeid Adorisio S., Tscholl P.M., Fucentese S. (2025). Current trends in the treatment of focal cartilage lesions: A comprehensive review. EFORT Open Rev..

[17] Milanovic F., Aksovic N., Bjelica B., Topalovic N., Arsenovic M. (2021). Patellar chondromalacia among adolescent athletes—A systematic review. Arch. Sports Med. Physiother..

[18] Bonnin M., Amendola A., Bellemans J., MacDonald S., Ménétrey J., Didden K., Bonnin M., Amendola A., Belemans J., MacDonald S.J., Ménétrey J. (2012). Patellofemoral osteoarthritis: Pathophysiology, treatment, and results. The Knee Joint: Surgical Techniques and Strategies.

[19] Vrgoč G., Vuletić F., Matolić G., Ivković A., Hudetz D., Bulat S., Janković S. (2023). Clinical outcome of arthroscopic repair for isolated meniscus tear in athletes. Int. J. Environ. Res. Public Health.

[20] Nakayama H., Kanto R., Kambara S., Kurosaka K., Onishi S., Yoshiya S., Yamaguchi M. (2017). Clinical outcome of meniscus repair for isolated meniscus tear in athletes. Asia-Pac. J. Sports Med. Arthrosc. Rehabil. Technol..

[21] Kim S.H., Park Y.B., Kim B.S., Lee D.H., Pujol N. (2021). Incidence of associated lesions of multiligament knee injuries: A systematic review and meta-analysis. Orthop. J. Sports Med..

[22] Shamrock A.G., Hall J.R., Hajewski C.J., An Q., Duchman K.R. (2022). Cartilage and meniscus injuries are more common in patients undergoing delayed multiligament reconstruction. J. Knee Surg..

[23] Marigi E., Keyt L.K., Laprade M.D., Camp C.L., Levy B.A., Dahm D.L., Stuart M.J., Krych A.J. (2021). Surgical treatment of isolated meniscal tears in competitive male wrestlers: Reoperations, outcomes, and return to sport. Orthop. J. Sports Med..

[24] Slauterbeck J.R., Kousa P., Clifton B.C., Naud S., Tourville T.W., Johnson R.J., Beynnon B.D. (2009). Geographic mapping of meniscus and cartilage lesions associated with anterior cruciate ligament injuries. J. Bone Jt. Surg. Am..

[25] Spitalul Clinic Județean de Urgență Arad Secția Clinică Ortopedie și Traumatologie. https://www.scjarad.ro/ortopedie-si-traumatologie/.

[26] Mordecai S.C., Al-Hadithy N., Ware H.E., Gupte C.M. (2014). Treatment of meniscal tears: An evidence-based approach. World J. Orthop..

[27] Sterne J.A., Hernán M.A., McAleenan A., Reeves B.C., Higgins J.P.T., Higgins J.P.T., Thomas J., Chandler J., Cumpston M., Li T., Page M.J., Welch V.A. (2019). Assessing risk of bias in a non-randomized study. Cochrane Handbook for Systematic Reviews of Interventions.

[28] Şuşan M., Cristea A.M., Drăghici G.A., Nica D.V., Florescu S., Damian C.G. (2025). Patterns of meniscal injuries in adults aged 35 and older: A retrospective analysis of surgical cases. Medicina.

[29] Ventura M., Seabra P., Oliveira J., Sousa P., Quesado M., Sousa H., Oliveira J. (2023). Meniscal injuries in patients aged 40 years or older: A comparative study between meniscal repair and partial meniscectomy. Cureus.

[30] Brittberg M., Winalski C.S. (2003). Evaluation of Cartilage Injuries and Repair. J. Bone Jt. Surg. Am..

[31] Georgescu M., Drăghici G.A., Oancea E.-F., Dehelean C.A., Șoica C., Vlăduț N.-V., Nica D.V. (2021). Effects of cadmium sulfate on the brown garden snail Cornu aspersum: Implications for DNA methylation. Toxics.

[32] Nica D.V., Bordean D.M., Pet I., Pet E., Alda S., Gergen I. (2013). A novel exploratory chemometric approach to environmental monitoring by combining block clustering with Partial Least Square (PLS) analysis. Chem. Cent. J..

[33] Luvsannyam E., Jain M.S., Leitao A.R., Maikawa N., Leitao A.E. (2022). Meniscus tear: Pathology, incidence, and management. Cureus.

[34] Fodor P., Sályom Á., Ivănescu A., Fodor R., Bățagă T. (2018). Prevalence of chondral lesions in knee arthroscopy. J. Interdiscip. Med..

[35] Çolak C., Naveen S., Bullen J., Ilaslan H. (2018). Incidence of medial meniscal tears in various age groups. J. Acad. Res. Med..

[36] Taunton J.E., Ryan M.B., Clement D.B., McKenzie D.C., Lloyd-Smith D.R., Zumbo B.D. (2002). A retrospective case-control analysis of 2002 running injuries. Br. J. Sports Med..

[37] Buchbinder R., Harris I.A., Sprowson A. (2015). Management of degenerative meniscal tears and the role of surgery. BMJ.

[38] Stoker D.J. (1980). Knee Arthrography.

[39] Nakamae A., Sumen Y., Tsukisaka K., Deie M., Fujimoto E., Ishikawa M., Adachi N. (2022). A larger side-to-side difference in anterior knee laxity increases the prevalence of medial and lateral meniscal injuries in patients with ACL injuries. Knee Surg. Sports Traumatol. Arthrosc..

[40] Makris E.A., Hadidi P., Athanasiou K.A. (2011). The knee meniscus: Structure–function, pathophysiology, current repair techniques, and prospects for regeneration. Biomaterials.

[41] Gee S.M., Tennent D.J., Cameron K.L., Posner M.A. (2020). The burden of meniscus injury in young and physically active populations. Clin. Sports Med..

[42] Giarmatzis G., Aggelousis N., Marinidis M., Fotiadou S., Giannakou E., Makri E., Vadikolias K. (2025). Asymmetric knee joint loading in post-stroke gait: A musculoskeletal modeling analysis of medial and lateral compartment forces. Biomechanics.

[43] Florescu S., Minda D., Damian C.G. (2025). First-time meniscal surgeries reveal age-linked rise in medial tears and sex-based injury difference. Appl. Sci..

[44] Yang G.Y., Guo H.L., Li T., Shang H.B., Zhao Y.F., Shi Y.Y. (2020). The medial compartment and patellofemoral joint degenerate more severely in early stage knee osteoarthritis: A cross-sectional study. Eur. Rev. Med. Pharmacol. Sci..

[45] Liu Y., Shah K.M., Luo J. (2021). Strategies for articular cartilage repair and regeneration. Front. Bioeng. Biotechnol..

[46] Sadeghi M.M., Açıkgöz E., Emans P., Zijta F., Tömer N., Tuijthof G., Roth A. (2025). Knee focal cartilage defect location heat map and local surface morphology characterisation: Insights for focal knee resurfacing implant design. J. Exp. Orthop..

[47] Vincent K.R., Conrad B.P., Fregly B.J., Vincent H.K. (2012). The pathophysiology of osteoarthritis: A mechanical perspective on the knee joint. PM&R.

[48] Kim M.S., In Y., Kim H., Jeong J., Sohn S. (2025). Why hoop tension matters: A biomechanical perspective on medial meniscus posterior root tears—A narrative review. Bioengineering.

[49] Hsu H., Siwiec R.M. (2024). Knee osteoarthritis. StatPearls [Internet].

[50] Kręcisz K., Bąk D., Krzemień-Szymanowska A. (2022). Using nonlinear vibroarthrographic parameters for age-related changes assessment in knee arthrokinematics. Sensors.

[51] Ding C., Cicuttini F., Scott F., Glisson M., Jones G. (2003). Sex differences in knee cartilage volume in adults: Role of body and bone size, age and physical activity. Rheumatology.

[52] Değirmenci E., Yücel İ., Özturan K.E., Karaduman Z.O., Karaca E. (2020). Evaluation of the age and gender-related changes in the Blumensaat line. Surg. Radiol. Anat..

[53] Hudelmaier M., Glaser C., Hohe J., Englmeier K.H., Reiser M., Putz R., Eckstein F. (2001). Age-related changes in the morphology and deformational behavior of knee joint cartilage. Arthritis Rheum..

[54] Al-Bayati Z., Coskun Benlidayi I., Gokcen N. (2018). Posture of the foot: Don’t keep it out of sight, out of mind in knee osteoarthritis. Gait Posture.

[55] Bottomley J., Al-Dadah O. (2023). Arthroscopic meniscectomy vs meniscal repair: Comparison of clinical outcomes. Cureus.

[56] Howell R., Kumar N.S., Patel N., Tom J. (2014). Degenerative meniscus: Pathogenesis, diagnosis, and treatment options. World J. Orthop..

[57] Atik I., Gul E., Atik S. (2024). evaluation of the relationship between knee osteoarthritis and meniscus pathologies. Malawi Med. J..

[58] Zech A., Hollander K., Junge A., Steib S., Groll A., Heiner J., Rahlf A.L. (2022). Sex differences in injury rates in team-sport athletes: A systematic review and meta-regression analysis. J. Sport Health Sci..

[59] Di Martino A., Barile F., D’Agostino C., Castafaro V., Cerasoli T., Mora P., Ruffilli A., Traina F., Faldini C. (2024). Are there gender-specific differences in hip and knee cartilage composition and degeneration? A systematic literature review. Eur. J. Orthop. Surg. Traumatol..

[60] Ahmed I., Radhakrishnan A., Khatri C., Metcalfe A.J. (2021). Meniscal tears are more common than previously identified; however, less than a quarter of people with a tear undergo arthroscopy. Knee Surg. Sports Traumatol. Arthrosc..

[61] Elkaïm M., Thès A., Lopes R., Andrieu M., Cordier G., Molinier F., Benoist J., Colin F., Boniface O., Guillo S. (2018). Agreement between arthroscopic and imaging study findings in chronic anterior talo-fibular ligament injuries. Orthop. Traumatol. Surg. Res..

[62] Sim J., Wright C. (2005). The kappa statistic in reliability studies: Use, interpretation, and sample size requirements. Phys. Ther..

